# The Middleware for an Exoskeleton Assisting Upper Limb Movement

**DOI:** 10.3390/s22082986

**Published:** 2022-04-13

**Authors:** Przemyslaw Strzelczyk, Krzysztof Tomczewski, Krzysztof Wrobel

**Affiliations:** The Faculty of Electrical Engineering, Automatic Control and Informatics, Opole University of Technology, 45-758 Opole, Poland; przemyslawstrzelczyk@gmail.com

**Keywords:** upper limb exoskeleton, middleware, rehabilitation, control system, simulation

## Abstract

This article presents the possibilities of newly developed middleware dedicated for distributed and modular control systems. The software enables the exchange of information locally, within one control module, and globally, between many modules. The executed information exchange system speed tests confirmed the correct operation of the software. The middleware was used in the control system of the active upper-limb exoskeleton. The upper-limb rehabilitation exoskeleton structure with six degrees of mechanical freedom is presented. The tests were performed using the prototype with three joints. The drives’ models of individual joints were developed and simulated. As a result, the courses of the motion trajectory were shown for different kinds of pressure on the force sensors, and different methods of signal filtering. The tests confirmed a correct operation of middleware and drives control system.

## 1. Introduction

Nowadays, powered exoskeletons can be used in various industries where heavy lifting or repeated and specific movements are required as a result of manufacturing activities [1,2,3]. Exoskeletons are also present and can be used in the arms [4,5], aerospace [6], and medical [7,8] industries. In medicine, these devices are used for both rehabilitation exercises and movement support [9,10,11,12]. There are numerous types of such devices, including those replacing individual limbs [13,14,15,16,17] or their parts [18,19,20,21] up to those replicating the entire human body [22,23,24]. An example of an exoskeleton used for rehabilitation might be the Lokomat [25,26], REX [10], or ReWalk [27]. Hardware and software configurations are tailored to specific dysfunctions [13]. Some of these devices are affixed to the patient’s limb or its fragment [28,29,30,31]. Others are mounted to wheelchairs or beds [32,33], depending on the specific condition.

Such devices are complex due to their control systems, among other things. Most have dedicated specialized software. One way to reduce the cost of such devices is to use generic software, requiring only partial modification to adapt to individual device characteristics [34,35].

Depending on their intended use, rehabilitation exoskeletons can serve three basic functions. The primary one is to support the patient’s motion. The control of such a device is frequently implemented based on force sensors [36,37] and position tracking is based on accelerometers and gyroscopes [38]. This feature is useful for conditions that impair the patient’s ability to move. It enables strengthening of movements and, optionally, limb trembling reduction by filtering sensor signals.

If the limb is completely unable to move, the exoskeleton can perform programmed movements set by a computer program [39].

The third function that rehabilitation exoskeletons can perform is to replace the physical therapist to some extent. In this mode of operation, the exoskeleton can introduce an appropriately selected load. This function can be used during rehabilitation exercises.

At the same time, the rehabilitation exoskeleton must meet a number of safety requirements. Its design should be mechanically and electrically protected against the possibility of exceeding the established ranges of motion in the joints, and the control system must allow the torque and speed in the joints to be limited to safe values, depending on the chosen rehabilitation method. A properly adjusted torque value prevents injury and allows to create resistance for the movement performed by the rehabilitated person. An example of this is the Proprioceptive Neuromuscular Facilitation (PNF) approach. Some tasks can be executed by the control system. It ensures that both control and safety functions are performed.

The control system can be built in a centralized or distributed form [36,40]. In exoskeletons, a centralized system requires running multiple control and power cables through movable joints, increasing the likelihood of failure. In the case of distributed systems, the control units are located at the kinematic joints, and individual power and communication buses are run between individual segments [36]. Additionally, using a distributed system, it is easier to develop a device in a form that can be freely configured from standard modules. However, a distributed control system greatly complicates the implementation of the control program and requires much more effort in developing the control software.

In order to simplify the implementation of control programs, it is possible to use middleware between the hardware and control applications, which would ensure the exchange of data between control modules [41]. In addition, the middleware may enable automatic configuration of the system and perform basic safety functions. This partial software unification reduces the time to develop control software and lowers the cost of application development. The software platform prepared in this way can be connected to developed applications that execute device-specific control algorithms.

The main goal of the project presented in this article was to develop a platform accelerating the implementation of control systems in currently ongoing projects. The advantage of this software to large systems is a simple way of usage in control programs while maintaining many functionalities offered by large systems. The currently developed software was used in the ongoing project of the upper limb exoskeleton.

The following issues are presented in the next sections of the article. Section 2 presents the structure of developed middleware dedicated for distributed and modular control systems, and the results of executed tests. Section 3 discusses the used structure of the six-joint upper limb rehabilitation exoskeleton, and the construction of a simplified three-joint prototype. Section 4 contains a description of the drives used in the prototype and a description of a simulation program used to determine the parameters of the controllers. Section 5 presents a distributed control system based on the developed middleware. Section 6 contains the measurement results of the prototype system in different operating states. Section 7 contains a discussion of obtained results.

## 2. Middleware

The middleware developed as part of this project forms the basis for the implementation of the control software and enables verification of the modules operation and automatic configuration of the control system. This makes it possible to reuse ready-made modules in future versions of the software, and in other projects requiring similar in-depth solutions. The modularity and genericity of the middleware eliminates the need to re-develop control software due to incompatibility, hardware platform change, operating system change, etc. [42].

The middleware included in the control software architecture separates the control applications from the hardware platform. If the hardware platform is modified or changed, the middleware allows you to avoid modifying the control applications. Examples of this type of software for large control systems include ROS and RT-Middleware [43,44]. However, their weaknesses for small systems are their excessive complexity and relatively long time required to configure the system.

The presented middleware is dedicated to small automation and robotic systems. The middleware is divided into five layers that cooperate with each other. The cooperation consists mainly of using mutually shared functionalities.

The control system that utilizes the middleware can be both centralized and distributed, requiring data exchange between multiple hardware units (instances). In order to realize local and distributed communication support, this middleware has two communication layers: LNCL (Local Node Communication Layer) and GNCL (Global Node Communication Layer) [45]. The former is responsible for communication between different parts of the middleware within an instance. From a software perspective, an instance means a single run of the main supervisory application that starts the middleware. The architectural assumptions of the presented middleware allow it to maintain only a single running monitoring application within a single hardware module and operating system.

The local communication mechanism is based on the exchange of information through messages. Communication is carried out between addresses registered in the layer using defined interface functions. Communication can take place between applications and threads but if required can also be performed within the same thread. The address registered in the layer is unique within a given network node. Its design consists of a 32-bit part specifying the node address and a 32-bit part representing the local address assigned by the LNCL mechanism. The data transmission is done using Unix domain sockets in streaming mode and is implemented in the shared memory area. A stream mode was chosen because of the preservation of the order of data delivery and the ability to retransmit lost message fragments.

In a system consisting of multiple middleware instances located in distributed network elements, the GNCL layer is used to transfer information between two instances [45]. This layer allows messages to be sent between the nodes of the system, i.e., the hardware modules. The control application, using the functionality of the global information exchange mechanism, defines employing a numerical identifier to which node a given message is to be delivered. If a local node is selected, messages are sent within the LNCL. If the identifier is different from that of the local node, the message is forwarded to the GNCL of the local node and then to the GNCL located at the destination node. Communication in the global layer is based on a TCP/IP protocol.

Another layer included in the developed middleware is the layer responsible for distributing user-defined events. An event can be any change in the state of the control system. This is, for example, a temporary loss of communication with a component critical for the correct execution of the control algorithm. When a loss of communication is detected, the system creates an event which, according to a predefined scenario, is propagated locally (to applications located in the same module) or globally (to applications located in other nodes). The event propagation function makes it possible to start procedures that allow for reacting to a random event and securing the correct operation of the control system, e.g., by initiating the operation of an alternative hardware component that can replace the basic module until communication is resumed. The software also enables the use of the Event Broadcast Layer (EBL) mechanism to replace typical message-based communication with event-based communication. The user has the ability to define the propagation range of a given message through an argument passed to the interface function.

The developed middleware is also equipped with an automatic module detection mechanism. The detection is done through mechanisms implemented in the Node Detection Layer (NDL) [45]. This layer allows to specify the list of available modules running under middleware control and provides node addresses. Utilizing the middleware, the control application can retrieve the current list of instances included in the system via an interface. Thanks to the cooperation of the EBL with the NDL, in case of a change in the system structure, e.g., in case of a loss of connectivity between modules, the predetermined applications will receive a notification about the link failure and, using the NDL functionality, they will be able to retrieve the list of modules with which the system currently maintains connectivity. This makes it possible to adjust the operation of the control algorithm while it is running in the event that part of the control system fails.

The presented middleware can be used in any project requiring communication and response to external events. An example of practical use of this functionality can be the reconfiguration of the system in case of damage to one of the manipulator joints. The provision of such information makes it possible to assume a fixed position of the damaged joint and modify the kinematic structure of the manipulator, which, in some cases, may allow the system to continue its operation, with limitations due to the loss of functionality of that joint.

**Figure 1:**
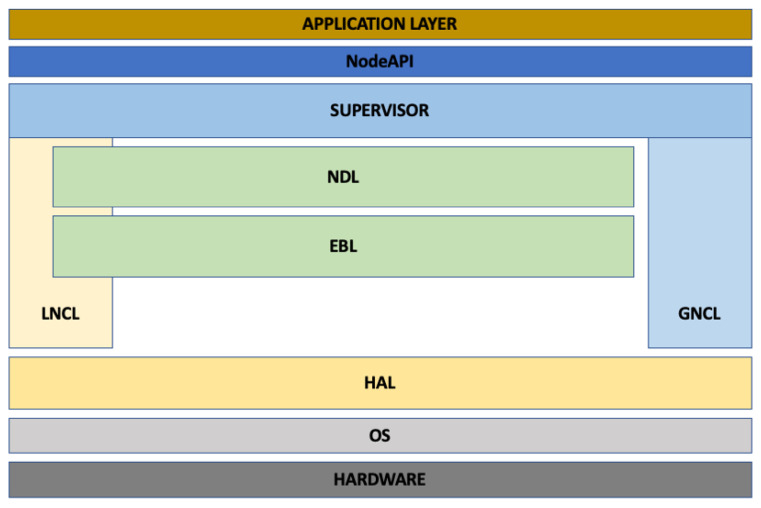
Schemes follow the same formatting.

An additional element of the developed middleware is a layer that controls the interaction of the control applications with the hardware installed in the module. This layer, through software abstraction, allows the API functions it provides to perform operations on hardware. This way, if a hardware component is changed, only the driver included in the middleware package needs to be modified. The control application code remains unchanged. This separation saves significant time when modifying a design. The adopted structure of the developed middleware is shown in Figure 1.

Descriptions in the Figure 1 mean, respectively:

1. APPLICATION LAYER—the layer of control applications that use middleware functionality, e.g., a program or set of programs that implement the assisted exoskeleton control algorithm;
2. NodeAPI—a library containing API functions that allow you to use the functionalities offered by middleware layers;
3. SUPERVISOR—the layer that oversees the runtime of individual middleware layers;
4. NDL (Node Detection Layer)—the layer that provides node detection functionality in a given network. Usage example: downloading of the current modules list connected to the network;
5. EBL (Event Broadcast Layer)—the layer that provides propagation functionality. Usage example: propagating information in selected levels of the control system and creating error and event handling procedures;
6. LNCL (Local Node Communication Layer)—the layer that provides communication mechanisms operating locally. Usage example: sending a message of any type to the selected recipient in the local control system;
7. GNCL (Global Node Communication Layer)—the layer that provides communication mechanisms operating globally. Usage example: sending a message of any type to the selected recipient in the distributed control system;
8. HAL (Hardware Abstraction Layer)—the abstract hardware layer;
9. OS—Operating System;
10. HARDWARE—the physical hardware layer.

### Information Exchange System Testing

Tests of the information exchange system were carried out for various configurations, including a system with four control units. The tests involved measuring data packet transfer times between test applications running on these modules.

The data exchange method is defined as follows. A random dataset of 256 MB is stored in RAM. Each of the running applications sends a 128 B packet, which is a consecutive portion of the generated random data, to the other three modules. Each GNCL packet sent is confirmed with a return message. After 256 MB of data has been transferred, the test is completed, and the time is measured. In order to present the change in system performance and the results obtained with the increasing size of individual packets, an additional test was performed with packets of 8192 B in size. Table 1 shows example results obtained for successive packets of 128 B and 8192 B sizes. The results presented in Table 1 come from the scenario “everyone-to-everyone”, which generates the biggest load on the tested system. The sub-score of the test is the averaged transmission time, from sending the request to receiving the acknowledgement of the packet receipt.

As a result of the tests conducted with data packets of 128 B, an average transfer rate of 0.82 Mbps was achieved. The average transfer time for a single packet is 587 μs. For the test with 8192 B packets, the average information exchange rate was 16.75 Mbps, and the averaged transfer time of a single packet was 1859 μs.

For 128 B packets, the data packet transfer cycle including acknowledgement was shorter than the assumed control time constant of 1 ms, which makes it possible to use the application to install the control program.

## 3. Implementation of the Environment in an Arm Exoskeleton Control System

**Figure 2:**
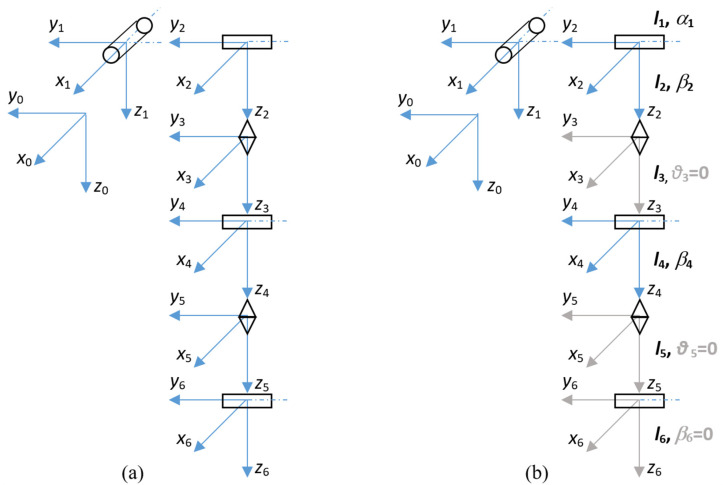
Structure of the exoskeleton: the full configuration on the left (**a**) and the structure of the constructed prototype on the right (**b**).

The control system includes the structure of an exoskeleton drive system to enable assisted movement of the upper limb, having six degrees of mechanical freedom. These are, respectively, the joints executing shoulder movements (two joints), arm twist, elbow flexion, forearm twist, and wrist flexion. The control program was adapted to control the established structure of the exoskeleton. A simplified structure with three mechanical degrees of freedom, implementing two shoulder movements and an elbow movement, is considered in the prototype system. Thanks to mechanisms implemented in the middleware, the control system automatically recognizes the configuration and adapts the control program to the current configuration. The full exoskeleton structure implemented in the software and the simplified prototype structure is shown in Figure 2. The prototype used for testing does not include the components responsible for arm and forearm rotation (2 and 4), and wrist flexion and extension 5. The control program only takes into account the length of these sections at a constant angular position relative to the preceding section. The missing modules in the prototype are highlighted in gray in the figure on the right.

The components highlighted in gray in Figure 2 were not installed in the prototype:

1. Rotary joint that performs adduction and abduction of the arm;
2. Rotary joint that performs flexion and extension of the arm;
3. Rotary joint that performs rotation of the arm;
4. Rotary joint that performs flexion and extension at the elbow;
5. Rotary joint that performs the rotation of the forearm;
6. Rotary joint that performs flexion and extension at the wrist.

The kinematic parameters of the system are summarized in Table 2. The center of the global coordinate system (*x*0, *y*0, *z*0) is assumed in the shoulder of the operator. Positive values of the angles include joint movements backward or outward from the trunk, while negative values include joint movements forward or toward the trunk. Table 3 shows the assumed maximum ranges of motion of each joint. These ranges are limited by mechanical stops.

Table 3 shows the uniform transformation matrices for each joint when detected and registered (left) and when not registered (right) in the information exchange system. The resulting matrix for the exoskeleton is calculated from (1). The symbols $s_{i}$ and $c_{i}$ denote the sine and cosine functions, respectively. The indexes used in the functions represent the angle variable in the joint i e.g., $s_{1}$ means $\sin\left(\alpha_{1}\right)$_._ $A_{i-1}^{i}$ is the transformation matrix of joint i.

(1) $$A_{6}^{0}=\prod_{i=1}^{6}A_{i-1}^{i}\;,$$

In the general case for six kinematic joints, the system has the following form:

$$A_{0}^{6}=A_{0}^{1}{}_{\theta_{1}}A_{1}^{2}{}_{\theta_{2}}A_{2}^{3}{}_{\alpha_{3}}A_{3}^{4}{}_{\theta_{4}}A_{4}^{5}{}_{\alpha_{5}}A_{5}^{6}{}_{\theta_{6}}$$

On the other hand, for the discussed prototype with three kinematic joints, according to Table 3, the automatic detection resulted in: $A_{0}^{6}=A_{0}^{1}{}_{\theta_{1}}A_{1}^{2}{}_{\theta_{2}}A_{2}^{3}{}_{0}A_{3}^{4}{}_{\theta_{4}}A_{4}^{5}{}_{0}A_{5}^{6}{}_{0}$.

For each joint in the exoskeleton, it was assumed that the range of motion could be limited, depending on the physiological characteristics of the user. The adopted permissible ranges of motion are presented in Table 4. Depending on the individual characteristics of the user, they may be limited. For this purpose, limit switches with adjustable position and software stops were additionally introduced.

The mechanical solutions used in the prototype were designed in CAD/CAM. The first component of the system is a joint that implements the arm abduction and adduction movements. The component consists of six fragments that form a fan set, allowing its fragments to be folded and unfolded, creating a gear with a ratio of 3.84:1. This component is driven by a 50 W brushless motor with an integrated planetary gearbox with a 66:1 ratio and an encoder. The joint consists of a gear attached directly to the output shaft of the gearbox. These components were printed using a 3D printer. The joint is equipped with a cam that activates limit switches depending on the position of the arm. The axis of rotation of this element is close to the natural position of the axis of rotation of the human shoulder.

Another component of the mechanical system is a joint that allows making flexion and extension movements of the arm. This movement is executed utilizing a bevel gearbox with a ratio of 1:1 and a planetary reduction gear attached to the motor. Both gearboxes are installed in series. The first one consists of two standard gears. A planetary gearbox with a 66:1 ratio is attached to the shaft of the 90 W brushless motor. This element is connected to the first one by a bearing body. This mechanism has two cams that activate limit switches, allowing you to set a limit on the range of motion.

The third component of the prototype’s mechanical system is a joint that allows the elbow joint to flex and extend. The connection between the second and third components is made using a dedicated forearm joint, mounted to the gear output of a 90 W motor. The forearm is driven by a brushless motor rated at 50 W. The drive is transmitted through two gearboxes connected in series, the first is a bevel gearbox with a ratio of 1:1 and the second is a planetary gearbox with a ratio of 43:1. The exoskeleton fragment from the joint to the wrist was made using a fragment of a rehabilitation orthosis. The fragment used was modified and adapted for this design.

The developed design allows for the installation of additional joints in the arm and forearm (rotation) and the wrist joint (flexion), according to the target structure shown in Figure 2.

**Figure 3:**
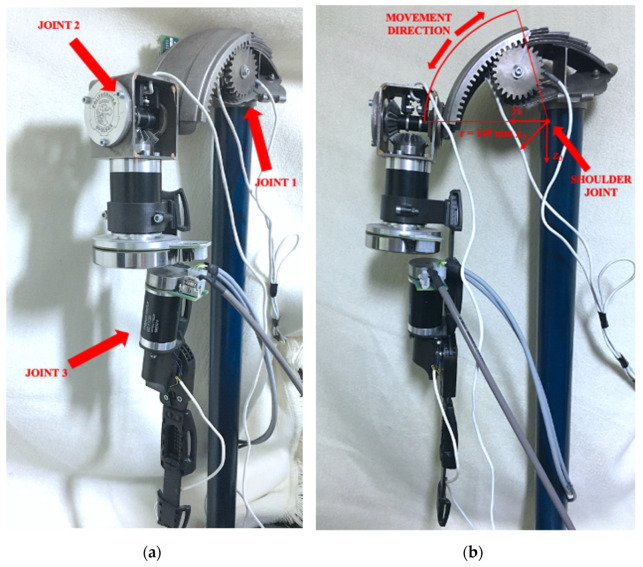
Prototype of an exoskeleton with three degrees of mechanical freedom: (**a**) side and (**b**) front view.

The prototype system used for testing was mounted on a support. The structure can be mounted on a wheelchair similar to [46]. The prototype exoskeleton system used to test the information exchange system is shown in Figure 3.

**Figure 4:**
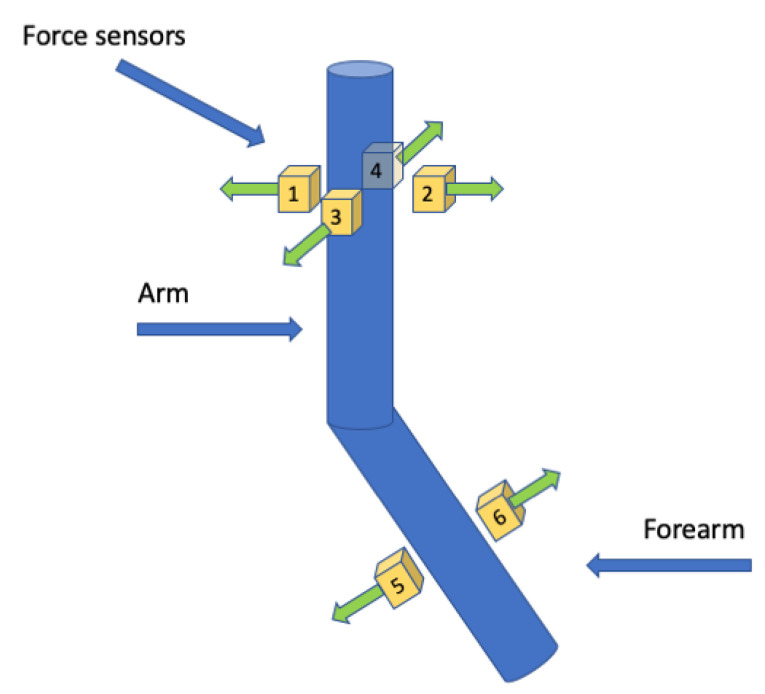
Distribution of force sensors (1–6) on individual components of the upper limb in the prototype system.

The prototype was adapted for control based on signals from six force sensors. The sensors were divided into two sets: the first intended to be mounted on the forearm, consisting of two force sensors responsible for flexion of the elbow joint, and the second intended to be mounted on the arm, consisting of four sensors responsible for abduction and adduction as well as flexion and straightening of the arm. The sensors were not attached to the exoskeleton structure when the system tests were performed. Force signals were applied by appropriately loading the individual sensors. The layout of the sensors in the prototype system is shown in Figure 4. To carry out full configuration, the number of sensors will be increased to detect twisting of the arm and forearm as well as flexion of the wrist.

Based on the values read from the sensors, the direction of movement of the individual members is determined. These values are sent to the inputs of the controllers. In order to allow the positioning of individual components when problems to hold the arm in a fixed position are encountered, minimum threshold values of control voltages were adopted, below which the signals at the inputs of the regulators are zeroed. This prevents system vibrations associated with the unstable position of the operator’s arm. In addition, the system was protected by introducing software limits on maximum torque and speed values. Due to the use of the exoskeleton for rehabilitation purposes, the speed was limited to low values. The adopted limits on acceleration are at about 30% of the drives’ capabilities. In addition, the system incorporates filtering of force sensor signals to prevent uneven drive operation caused by tremor of the operator’s upper limb.

**Figure 5:**
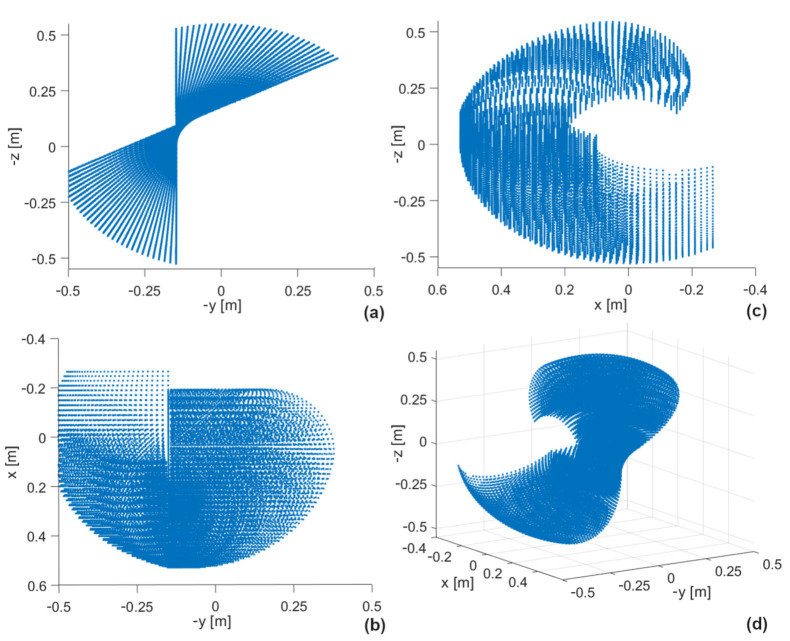
Workspace of the exoskeleton mechanical system (projections in three planes: (**a**) *zy*, (**b**) *xy*, (**c**) *zx* and (**d**) *3D*).

The kinematic model of the mechanical system was implemented in Matlab. Here, the coordinates occupied by the tip of the forearm were determined when all joints were moved over fixed ranges with a resolution of 1 deg. The simulation resulted in the workspace shown in Figure 5. In the main module, it is possible to limit this space by applying additional conditions. Figure 5 shows the projections on the ZY, ZX, and XY planes. The lower right figure shows the 3D workspace.

## 4. Determination of the Drive Parameters

Because of the limits imposed on speed and torque due to the exoskeleton’s intended use for rehabilitation purposes, drive tests and controller parameter selection were performed separately for the drive of each joint, ignoring the effect of the motion of other joints. The simulation model was developed based on the output characteristics of the drives as published by the manufacturer.

A simplified simulation model was adopted consisting of a BLDC drive, load, and PID controller modules. A simplified drive model based on the output characteristics of a complete drive unit with a mechanical transmission was used. The load module considers the losses in the rotational motion of the joint, the moment of inertia of the tested member, and the dependence of the force of gravity on the angular position of the member [47]. Module three contains a PID controller with a parallel structure.

**Figure 6:**
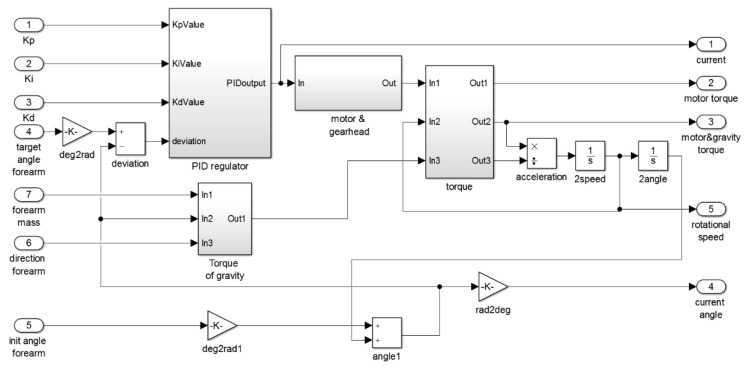
Simulation model of the elbow joint drive.

**Figure 7:**
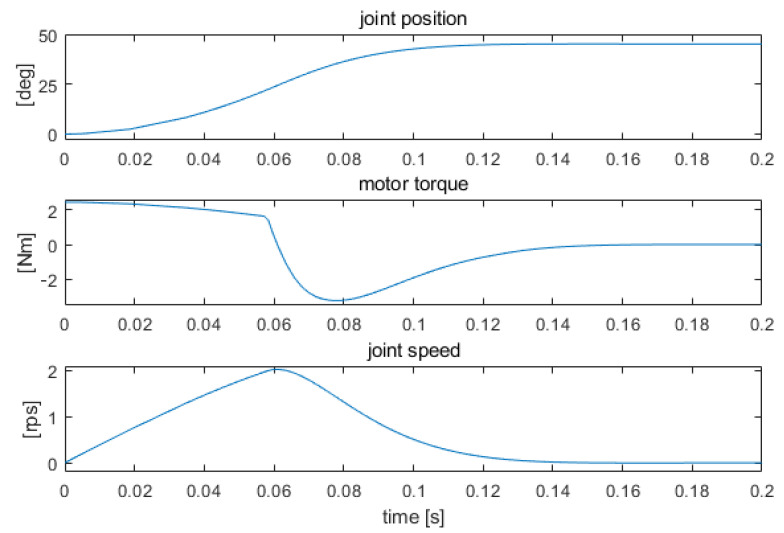
The course of a 45-degrees change in the position of the shoulder hinge.

Using a simulation model, the dependence of the controller parameters on the position of the manipulator members relative to the rest position along the torso was determined, minimizing the times of reaching the set position in the absence of overshoot. In this way, the maximum possible values of speed, torque, and acceleration at individual kinematic joints were determined. Actual velocity and torque values adjusted to the operator’s capabilities must be within this range, with significantly lower values for rehabilitation applications. The obtained relations were approximated and implemented in control programs in the form of quasi-adaptive parameters of PID controllers. The structure of the simulation model in Matlab Simulink and the examples of simulation results are shown in Figure 6 and Figure 7. It was assumed that the pressure sensor responsible for indicating movement intention of the desired direction was maximally actuated for the entire simulation run. In fact, the speed is dependent on the force sensor pressure, of which the level is dependent on the hand movement.

## 5. Control System

**Figure 8:**
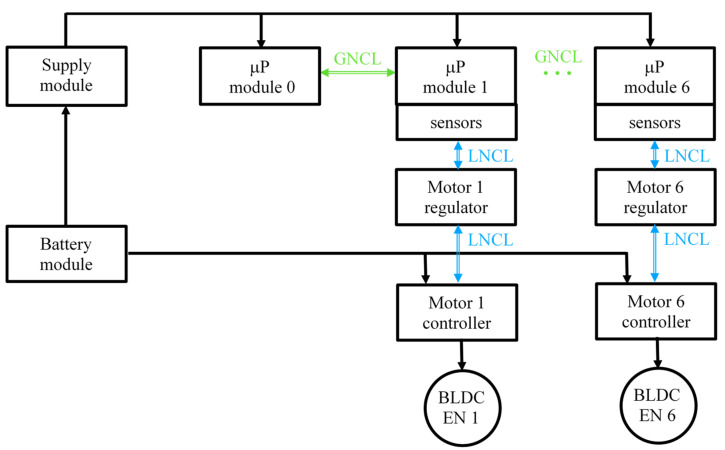
A structure of the six-joint exoskeleton control system.

The control system consists of three Raspberry Pi 2 hardware modules that control the operation of the drives. One of the modules acts as the main control unit. Its task is to control the current circuit structure and run control programs. In case of failure of this module, another module can take over this function. Communication between modules is based on Ethernet. A control program based on the developed middleware was run in each hardware module. A structure of the control system is shown in Figure 8. It comprises six modules that control the joints and a main module.

The control system features an automatic system for detecting and monitoring the presence of system components. The structure of the control system replicates that of the exoskeleton’s drive system. Each of the installed control programs is responsible for operating one joint. When using the described test prototype with an incomplete configuration, the system identifies three installed members and limits the control range to two joints at the shoulder and one at the elbow. Autoconfiguration of the system takes place in such a way that when the system is working, the programs implementing control algorithms receive information about the current state and the number of connected modules and then make appropriate corrections in their operation, taking into account the data provided. In the case presented, the control system will start to execute the control for the three installed members in the prototype system, omitting the undetected members. Autoconfiguration is made possible by the functionality offered by the EBL.

An example of the use of this functionality is when the control software detects that one of the three prototype modules has stopped responding to requests from the main central module while performing its tasks. In this case, the module is marked as faulty. The information that this module is unavailable is propagated through the EBL. When it is passed to the other modules, the control system is reconfigured, an attempt is made to recover the device, or the control algorithm is reconfigured. Reconfiguration of the control system involves considering the absence of a given module in the kinematic model of the system. The possibility of further operation of the system is conditioned by the limitations implemented in the control program.

In practice, with the help of the functionalities of the middleware, it is possible to freely define scenarios for handling the occurrence and propagation of an event in the control system. Such a scenario involves stimuli that activate a particular behavior (EBL event number) and a description of the behavior of the system when activated in the control program. The use of this middleware functionality allows the circuit structure to be automatically updated in the central control unit. In the prototype system, the control modules are responsible for, respectively, adduction and abduction of the arm, extension and flexion of the arm, as well as extension and flexion of the forearm. The main control unit is responsible for controlling the current system configuration and initializing the control programs in each registered module. The speed and torque limits of the drives in the joints, the limits of the ranges of motion, and the control of the drives are carried out by each of the control modules of the respective joint.

The control system in the prototype operates based on signals from force sensors. Because the structure of the prototype system is limited to three joints, signals from six sensors are actively used.

## 6. System Performance Tests

Performance tests of the system were carried out by stimulating the force sensors in different ways. During the tests, drive parameters were measured at continuous constant force, at intermittent force, and at simulated arm tremor that often occurs in the case of disease. According to the intended use of the exoskeleton, limits on maximum torque and speed values were adopted.

**Figure 9:**
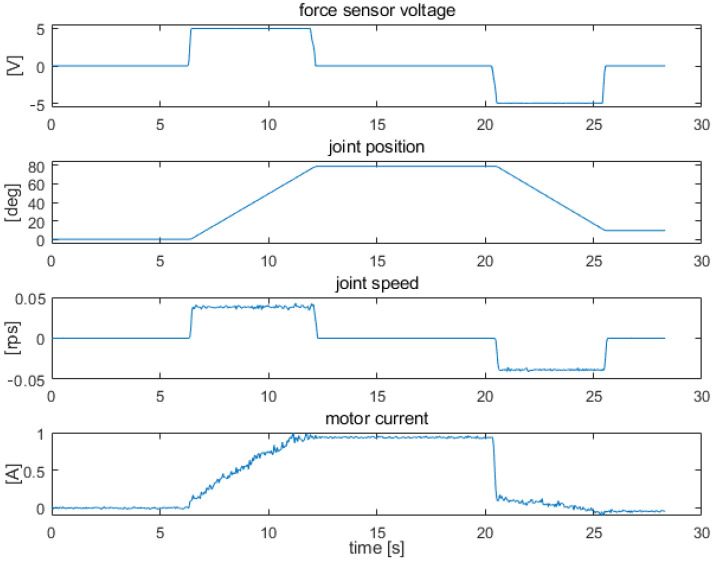
Signal waveforms from force, position, speed, and supply current sensors for elbow joint drive under strong constant force on the sensors.

**Figure 10:**
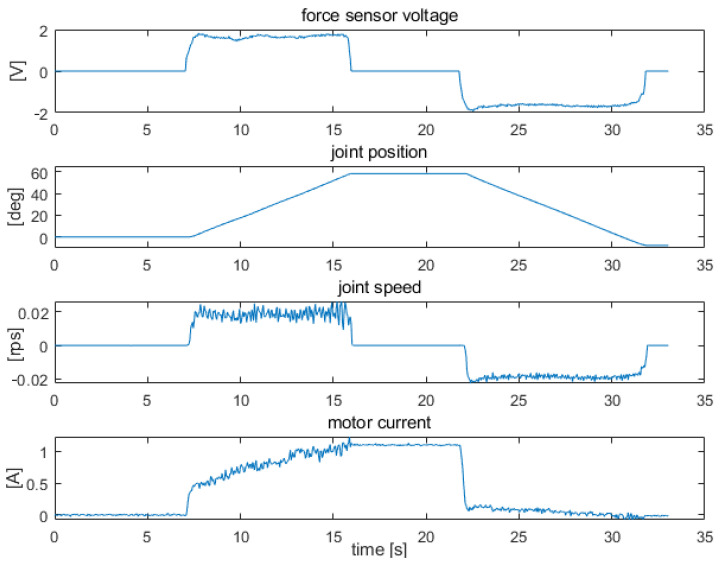
Signal waveforms from force, position, speed, and supply current sensors for elbow joint drive under light force on the sensors.

**Figure 11:**
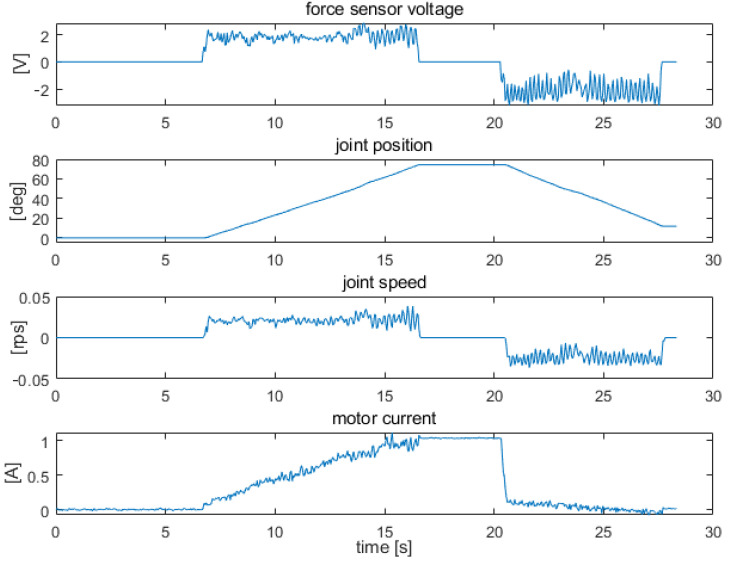
Signal waveforms from force, position, speed, and supply current sensors for elbow joint drive under uneven force on the sensors.

Figure 9, Figure 10 and Figure 11 show examples of measurements taken for the elbow drive. The tests consisted of measuring the patterns of the joint variables during the stimulation of the force sensors. Measurements were taken from a resting position (arm placed vertically along the trunk) to a position perpendicular to the trunk, with a range of approximately 90 deg. Tests were performed for the exoskeleton with an additional 0.5 kg load placed at the end of the forearm. The figures show, respectively, the measurement results obtained with strong constant force on the sensors, which caused saturation of the signals (Figure 9), with constant lower force on the sensors, which did not cause saturation of the signals (Figure 10), and with uneven force on the sensors, which simulated arm trembling (Figure 11).

The waveforms in Figure 9 show the behavior of the system when the sensors are pressed hard, causing saturation of the signals. Initially (up to 7 s), a resting position parallel to the trunk is maintained. The drive is not powered. Between 7 s and 12 s, a force signal is applied and the forearm is lifted at a constant speed to a position near perpendicular to the trunk. Then, between 12 s and 20 s, the arm is held in a fixed position. The condition of positioning is to maintain a low force of force on the sensors, within the assumed insensitivity range. From 20 s to 25 s, there is strong force on the sensor located on the opposite side, which lowers the forearm near the resting position. Both moves (down and up) were made at the maximum speed allowed by the introduced speed limits. The maximum speed is reached at the acceleration resulting from the adopted maximum torque limit.

In the second case (Figure 10), constant force was maintained on the sensors at a level that did not saturate the signals. As in the previous case, the movement was started from the rest position with the drive turned off. Between 7 s and 16 s the sensor responsible for forearm flexion was stimulated, and between 22 s and 32 s, the sensor responsible for forearm extension was stimulated. During the test, the forearm position was changed over a range of approximately 90 deg. The speed waveform shows slight pulsations due to unstable force on the sensors. The level of these pulsations when a stable force test is performed on the sensors varies from a dozen to several dozen percent. This can cause discomfort for the user even when the exoskeleton is controlled by a healthy person.

The example shown in Figure 11 illustrates the actuator operation in the case of uneven force on the sensors, which is caused by arm tremor. The tremor was largely transferred to control signals from the force sensors. The measurement conditions were similar to those presented earlier. The movement started from a resting position along the torso with the drive off. Between 7 s and 17 s, the sensor responsible for forearm flexion was activated, and between 21 s and 28 s, the sensor responsible for forearm extension was activated. The movements were performed over a range of approximately 90 deg. Large-amplitude pulsations are seen on the speed waveforms. The unevenness of motion is also clearly visible on the angular position waveform of the joint. Because of substantial speed fluctuations due to unstable force on the sensors, filtering of the force sensor signals was applied to the control system in the next stage of testing.

In the measurement system, the force sensor signal is a signal that oscillates around some expected value. This is due to the very movement of the exoskeleton arm, which keeps up with the movement of the human upper limb. Since the developed exoskeleton design is intended to be used for rehabilitation, people using this equipment may have various motor dysfunctions (e.g., Parkinson’s disease). Therefore, it is necessary to use a filter element in the measurement path to filter the signal from the force sensor.

The tests were conducted using two filtering methods: ongoing signal averaging and the Kalman filter. To evaluate the effect of filtering the sensor signals on the waveforms of position and speed, the force signal waveform is shown in Figure 3, which contains the pulsations with the largest amplitude, was filtered. The filter parameters and insensitivity levels were adjusted so that the delays introduced by the filters did not exceed 0.3 s. This is the value of the exoskeleton’s movement delay relative to arm movement that does not cause operator discomfort.

The averaging filter was defined as:

(2) $$\bar{x}=\frac{\sum_{a=-\left(n-1\right)}^{0}x_{a}}{n}$$

where $\bar{x}$ is the averaged value from *n* samples, measured at time Δ*t*. The number of samples *n* is determined by the set averaging time Δ*t*, which was set at 0.3 s, and $x_{0}$ is the last read sample.

**Figure 12:**
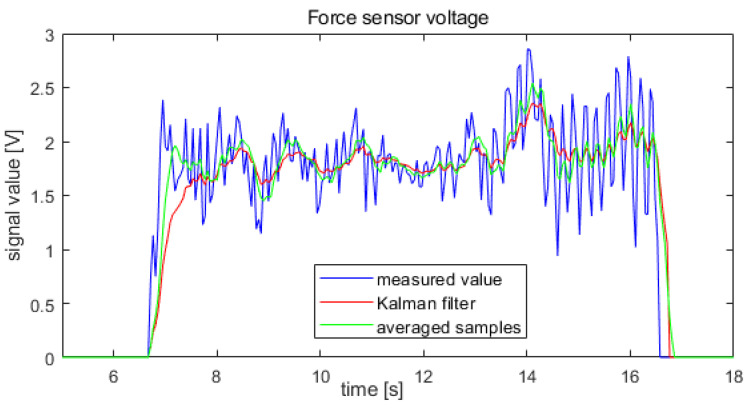
Signal waveforms from the force sensor during arm tremor: without filtration, after running averaging for 0.3 s, and after applying the Kalman filter.

The performance of the two filters was compared using the force sensor signal waveform shown in Figure 11. The results obtained using both filtration methods are presented in Figure 12.

The Kalman filter resulted in a greater reduction in the pulsation of the force sensor signal. Therefore, further tests of the system were conducted for a system with the Kalman filter.

**Figure 13:**
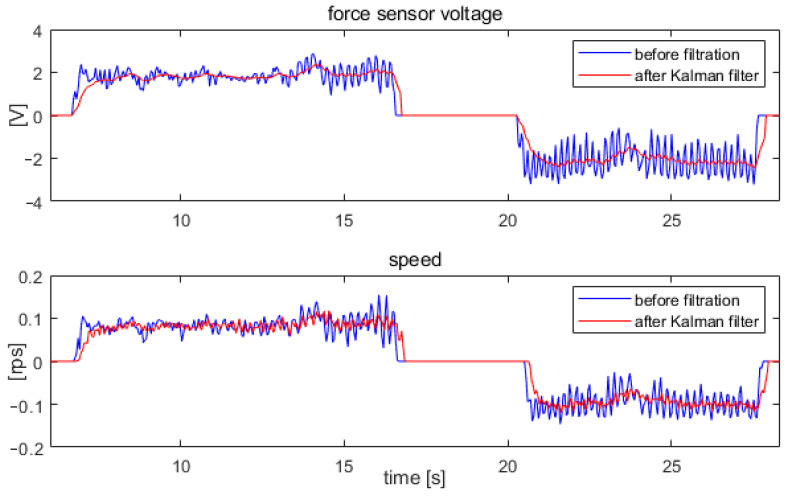
Signal waveforms from the force sensor and after applying the Kalman filter, and the corresponding speed waveforms at the elbow joint.

In the next step, the waveform obtained after filtering using the Kalman filter, shown in Figure 12, was used as the force signal. Thus, the waveforms of forearm position and speed were measured for the forcing signal shown in Figure 11 after applying the Kalman filter. A comparison of the velocity waveforms obtained with no filtering of the force sensor signals and those obtained with the Kalman filter is shown in Figure 13.

As a result of the Kalman filter, a significant reduction in drive speed ripple and exoskeleton vibration was achieved. After applying the Kalman filter, a reduction of speed pulsations to a level similar to that shown in Figure 9 was achieved when the force sensor signals were saturated.

## 7. Discussion and Conclusions

As part of the project, an information exchange system characterized by high universality and the possibility of easy implementation in various projects was developed. The system is equipped with mechanisms for automatic configuration and control of module functionality. It can be implemented in both modular and distributed control systems. The presented information exchange system was developed to accelerate the implementation of current and future projects. The system is so universal that it can be used, for example, to control a swarm of drones.

In the example shown, an information exchange system was used to control the drives of an active arm exoskeleton. The performed information exchange speed tests using 128 B packets demonstrated that the information exchange system can be used to control an exoskeleton with a given time constant of 1 ms in systems of much higher complexity. The tests carried out showed also that the system can be used in these types of projects, which was the main goal of the work.

During the project, a program to control a three-joint upper limb exoskeleton and a simplified prototype of an active arm exoskeleton were developed. The control system of the prototype was based on Raspberry Pi modules. Programs that control kinematic joint drives are based on signals from force sensors. The control programs were implemented using the mechanisms incorporated in the developed information exchange system. Each hardware module supports one drive. The programs implemented in the individual drive modules differ mainly in their controller parameters, ranges of motion, speed and torque limits, and the procedure for detecting the absence of any module.

Each module that is part of this control system can obtain information about the status of sensors of another module that is equipped with such sensors if this information is needed for the control algorithm implemented on a given module. The exoskeleton prototype for the experimental study was made in a simplified version containing two joints at the shoulder and one at the elbow, which are controlled by six force sensors, while the twisting of the arm and forearm as well as the flexion and extension of the wrist were omitted. The program implemented in the main control unit allows to control the position of individual joints and the effector, and to check whether the drives are working correctly. The check of the joints and effector position of the exoskeleton is based on the kinematic model obtained by the automatic configuration of the system from the registered modules.

The tests performed demonstrated that the information exchange system and the arm exoskeleton prototype worked properly based on force sensor signals. The use of force sensor signal filtering significantly reduced the transmission of arm tremor to the motion of the exoskeleton. The filter parameters were specifically chosen so as not to introduce delays in the movement of the exoskeleton that would be perceptible to the operator. For both filters (averaging and Kalman), an acceptable delay of 0.3 s was assumed. Using the Kalman filter, greater tremor reduction was achieved than using ongoing signal averaging. The tests of the prototype showed the correct operation of the control system based on the developed middleware. The design of the exoskeleton will be modified based on the results of tests with the participation of the operator.

In the next stage of testing, the prototype system will be equipped with missing arm and forearm twist joints and a wrist joint. Lower power drive units will also be used to reduce the weight of the system.

## Figures and Tables

**Table 1:** Results of data transmission time measurement in the developed information exchange system.

No.	Average Time to Transmit a 128B Packet (μs)	Average Time to Transmit an 8192B Packet (μs)	No.	Average Time to Transmit a 128B Packet (μs)	Average Time to Transmit an 8192B Packet (μs)
1	585	1891	6	582	1807
2	597	1871	7	596	1729
3	586	1853	8	601	1767
4	991	1964	9	609	1743
5	619	1871	10	596	1759

**Table 2:** Joint parameters of the upper limb exoskeleton.

No.	*θ*	*d*_i_	*β*_i_	*b*_i_	*α*_i_	*a*_i_
e1				*l*_1_	*α*_1_	
2		*l*_2_	*β*_2_			
3	*Θ*_3_	*l*_3_				
4		*l*_4_	*β*_4_			
5	*Θ*_3_	*l*_5_				
6		*l*_6_	*β*_6_			

where: *l*_1_—distance between shoulder joint hinges (14.9 cm); *l*_3_ + *l*_4_—arm length (28 cm); *l*_5_ + *l*_6_—forearm length (24 cm); *Θ*_i_, *β*_2_, *α*_1_—rotation angles around the *Z*, *Y*, *X* axes, respectively; *d*_i_, *b*_i_, *a*_i_—the length of the manipulator members, along the *Z*, *Y*, *X* axes.

**Table 3:** Transformation matrices for kinematic joints registered and unregistered in the system.

No.	Registered Joint	Unregistered Joint
1	$A_{0}^{1}{}_{\theta_{1}}=\left[\begin{array}{cccc} 1 & 0 & 0 & 0\\ 0 & c_{1} & -s_{1} & c_{1}l_{1}\\ 0 & s_{1} & c_{1} & s_{1}l_{1}\\ 0 & 0 & 0 & 1 \end{array}\right]$	$A_{0}^{1}{}_{0}=\left[\begin{array}{cccc} 1 & 0 & 0 & 0\\ 0 & 1 & 0 & l_{1}\\ 0 & 0 & 1 & 0\\ 0 & 0 & 0 & 1 \end{array}\right]$
2	$A_{1}^{2}{}_{\theta_{2}}=\left[\begin{array}{cccc} c_{2} & 0 & s_{2} & 0\\ 0 & 1 & 0 & 0\\ -s_{2} & 0 & c_{2} & l_{2}\\ 0 & 0 & 0 & 1 \end{array}\right]$	$A_{1}^{2}{}_{0}=\left[\begin{array}{cccc} 1 & 0 & 0 & 0\\ 0 & 1 & 0 & 0\\ 0 & 0 & 1 & l_{2}\\ 0\; & 0 & 0 & 1 \end{array}\right]$
3	$A_{2}^{3}{}_{\alpha_{3}}=\left[\begin{array}{cccc} c_{3} & -s_{3} & 0 & 0\\ s_{3} & c_{3} & 0 & 0\\ 0 & 0 & 1 & l_{3}\\ 0 & 0 & 0 & 1 \end{array}\right]$	$A_{2}^{3}{}_{0}=\left[\begin{array}{cccc} 1\; & 0 & 0 & 0\\ 0\; & 1 & 0 & 0\\ 0\; & 0 & 1 & l_{3}\\ 0\; & 0 & 0 & 1 \end{array}\right]$
4	$A_{3}^{4}{}_{\theta_{4}}=\left[\begin{array}{cccc} c_{4} & 0 & s_{4} & 0\\ 0 & 1 & 0 & 0\\ -s_{4} & 0 & c_{4} & l_{4}\\ 0 & 0 & 0 & 1 \end{array}\right]$	$A_{3}^{4}{}_{0}=\left[\begin{array}{cccc} 1 & 0 & 0 & 0\\ 0 & 1 & 0 & 0\\ 0 & 0 & 1 & l_{4}\\ 0 & 0 & 0 & 1 \end{array}\right]$
5	$A_{4}^{5}{}_{\alpha_{5}}=\left[\begin{array}{cccc} c_{5} & -s_{5} & 0 & 0\\ s_{5} & c_{5} & 0 & 0\\ 0 & 0 & 1 & l_{5}\\ 0 & 0 & 0 & 1 \end{array}\right]$	$A_{4}^{5}{}_{0}=\left[\begin{array}{cccc} 1 & 0 & 0 & 0\\ 0 & 1 & 0 & 0\\ 0 & 0 & 1 & l_{5}\\ 0 & 0 & 0 & 1 \end{array}\right]$
6	$A_{5}^{6}{}_{\theta_{6}}=\left[\begin{array}{cccc} c_{6} & 0 & s_{6} & 0\\ 0 & 1 & 0\; & 0\\ -s_{6} & 0 & c_{6} & l_{6}\\ 0 & 0 & 0 & 1 \end{array}\right]$	$A_{5}^{6}{}_{0}=\left[\begin{array}{cccc} 1 & 0 & 0 & 0\\ 0 & 1 & 0 & 0\\ 0 & 0 & 1 & l_{6}\\ 0 & 0 & 0 & 1 \end{array}\right]$

**Table 4:** Motion ranges of the prototype for the assumed base position.

Joint Identification	Joint 0	Joint 1	Joint 3
Motion range (degrees, min, max)	from 0 to 60	from −30 to 170	from 0 to 140
